# Clinical characterization and management of persistent genital arousal disorder/genito-pelvic dysesthesia (PGAD/GPD): a registry study

**DOI:** 10.1093/sexmed/qfaf106

**Published:** 2026-01-31

**Authors:** Franziska Maxi Lisa Marie Kümpers, Sophie Köhne, Tillmann H C Krüger

**Affiliations:** Department of Psychiatry and Psychotherapy, Medical Faculty, Heinrich Heine University Düsseldorf, 40629 Düsseldorf, Germany; Division of Clinical Psychology & Sexual Medicine, Department of Psychiatry, Social Psychiatry and Psychotherapy, Hannover Medical School, 30625 Hannover, Germany; Division of Clinical Psychology & Sexual Medicine, Department of Psychiatry, Social Psychiatry and Psychotherapy, Hannover Medical School, 30625 Hannover, Germany; Division of Clinical Psychology & Sexual Medicine, Department of Psychiatry, Social Psychiatry and Psychotherapy, Hannover Medical School, 30625 Hannover, Germany; Center of Systems Neuroscience, 30559 Hannover, Germany

**Keywords:** persistent genital arousal disorder (PGAD), genito-pelvic dysesthesia, sexual arousal, psychiatric comorbidities, urogynecology, MRI

## Abstract

**Background:**

Persistent genital arousal disorder (PGAD)/genito-pelvic dysesthesia (GPD) is a disabling disease, where patients perceive prolonged genital arousal without sexual desire. The condition mainly occurs in women. Etiopathological considerations reach from peripheral to central nervous system mechanisms.

**Aim:**

To clinically and anamnestically characterize patients with PGAD/GPD using data from a dedicated patient registry.

**Methods:**

This study comprises a detailed description of 92 patients with PGAD/GPD from a registry data bank. Investigations included clinical characterization of PGAD/GPD-symptoms, assessment of sexual, urogynecological, somatic, and psychiatric history as well as clinical examination and treatments.

**Outcomes:**

The primary outcome was to identify common clinical features, symptom patterns, trigger and relieving factors, comorbidities, and therapeutic strategies.

**Results:**

Persistent genital arousal disorder symptoms were mostly characterized as tingling and were almost permanently present. In over 80%, PGAD symptoms were located in the clitoris (women) or in the glans penis (men); 50% reported extragenital manifestations. Thirty-four percent described symptoms such as GPD. PGAD presented with high rates of swelling of the genitals (46%), spontaneous orgasms (30%), and extraordinary lubrication (27%). Most frequent trigger factors were mental stress, sitting, wearing tight clothes, and vibration. Relieving factors were mainly distraction, physical exercise, and warmth. Half of the patients stated increased urinary urge. More than 40% stated symptoms of overactive bladder syndrome. About one third reported restless legs symptoms. Almost 70% had comorbid psychiatric diseases, mainly depressive disorders. In most cases, those occurred after the onset of PGAD/GPD symptoms. Further diagnostic procedures covered urogynecological and neurological examinations as well as magnetic resonance imaging of brain, spinal cord, and pelvis. Non-pharmacological therapeutic approaches included among others physiotherapy, psychotherapy, transcutaneous electrical nerve stimulation, neurosurgical procedures, or pudendal block. In the majority, a clear somatic correlate for PGAD/GPD was not found.

**Clinical Implications:**

The findings highlight the complex and multifactorial nature of PGAD/GPD, advocating interdisciplinary diagnostics and individualized treatments due to high psychiatric comorbidity and the absence of consistent somatic findings.

**Strengths and Limitations:**

The study’s strength lies in its large sample size and comprehensive clinical profiling of PGAD/GPD patients; however, its retrospective registry-based design and the absence of a control group represent significant limitations.

**Conclusion:**

This first registry-based study of PGAD/GPD in a larger cohort highlights the need for future controlled studies with larger sample sizes and more specified clinical assessments according to consensus statements for a better understanding of the clinical appearance and the etiopathology of PGAD/GPD.

## Introduction

Persistent genital arousal disorder (PGAD) is a disturbing and undesirable syndrome which is not related to sexual pleasure. It was first defined by Leiblum and Nathan in 2001^1^ with the following 5 diagnostic criteria: (1) persistence of involuntary genital arousal over an extended period of time (>3 months), (2) it is not resolved after one or more orgasms, (3) there is no relation to subjective sexual desire or feelings, (4) it is not only triggered by sexual activity, but also by non-sexual stimuli or released without any obvious triggers, (5) the arousal is experienced as unwanted and intrusive and is correspondingly associated with distress.^1^^,^^2^ This definition goes hand in hand with the consensus paper of the International Society for the Study of Women’s Sexual Health of Goldstein and his colleagues.^3^ The authors extended the definition to include the primary localization at the clitoris or even at another genito-pelvic region. Furthermore, the association with orgasms was emphasized in the form of either being on the verge of an orgasm or to experience an excessive number of orgasms. On top, the syndrome was extended with the name genito-pelvic-dysesthesia (GPD), which is defined as an unpleasant and atypical sensation in the genito-pelvic region with a symptom quality of tingling, burning, pain, or itching. This definition also includes lower extremity dysesthesia due to a possible involvement of sacral nerve roots.^3^

The symptoms can be continuously present or intermittent and can be acquired or even lifelong in around 10%.^4^^,^^5^ Individuals who acquired the symptoms typically do so around the age of 37 and ~25% experience their symptom onset before the age of 18.^4^ The reported prevalence of PGAD/GPD is higher in women, but also men and even children can be affected.^1^^,^^3^^,^^4^^,^^6–9^ Estimations on the prevalence range from 0.5% to 6.7% without any available valid data.^3^^,^^5^^,^^8^^,^^10–12^ Etiopathological considerations reach from peripheral factors, such as pudendal nerve pathologies, polyneuropathy, and pelvic venous insufficiency,^10^^,^^13–26^ to central nervous system mechanisms including sacral Tarlov cysts, intervertebral disc pathologies and possible overlaps with restless legs syndrome (RLS), overactive bladder syndrome (OAB), or pharmacological influences^3^^,^^19^^,^^27–31^). For detailed summaries, see eg^32^ It is also discussed, whether psychological and social factors should be considered as a cause, a comorbidity or a consequence of PGAD/GPD. The disorder can be associated in particular with depression, anxiety, panic disorders, obsessive-compulsive disorders, trauma, and suicidality.^3^^,^^5^^,^^10^^,^^13^^,^^19^^,^^24^^,^^33–39^ For neuropsychopharmacological influences and findings of investigations of brain activity using functional magnetic resonance imaging (MRI) on PGAD/GPD patients see Kruger et al.^40–43^

The ongoing discourse concerning the etiology, pathophysiology, and potential mediators of PGAD/GPD is indicative of the prevailing uncertainty surrounding the condition. Most of the insights regarding PGAD/GPD are based on level III evidence, which means they rely on non-experimental, descriptive studies, and case studies. Hence, there is still an indispensable need for further research. Persistent genital arousal disorder is recognized in the ICD-11 under “other specified sexual arousal dysfunctions” (HA01.Y), underscoring its clinical relevance and the need for continued research on this condition. Based on our first systematized and controlled study with 52 subjects (26 PGAD/GPD patients and 26 healthy controls),^32^ our aim was to confirm the main results of our study with more patients out of an own register and to generate further insights into diagnostic and therapeutic procedures. Thus, we initiated a detailed description of the clinical features, comorbidities, diagnostic, and therapeutic approaches of PGAD/GPD patients.

## Methods

### Subjects and procedure

This study includes a detailed description of n = 92 patients with PGAD/GPD from a registry data bank at [Institution removed for double blind-review]. The analyzed data set consist of n = 86 women and n = 6 men. Of the collective of 92 subjects, 26 took part in the “iPGAD study” (see^32^).

The registry data bank includes all patients being treated for PGAD/GPD at MHH between January 2010 and September 2024 in our outpatient department. As part of the admission contract to our consultation for PGAD/GPD, subjects also consented to the use of the clinically collected data for scientific purposes. Data were collected in routine clinical practice and were anonymized for data analysis. The study was conducted in accordance with the Declaration of Helsinki 1964, updated in October 2024. The Ethics Committee of [Institution removed for double blind-review] confirmed in an official statement that, according to the professional code of conduct for physicians in the state of [name removed for double blind-review], an ethical review is not needed for retrospective anonymized data.

Inclusion criteria for subjects were defined as follows: female or male gender, every age, proficiency in German language, no acute and severe mental or somatic disease requiring immediate treatment. In addition, PGAD/GPD patients should fulfill the criteria of Leiblum and Nathan (2001, see criteria above). Subjects with severe intelligence impairment, acute physical or mental illness (eg, acute psychosis, brain damage, Alzheimer’s disease, severe bacterial infection) were excluded from the analysis. As none of these exclusion criteria were present in our registry data bank, no subjects needed to be excluded.

### Measures

Investigations included variables for the following domains: (1) clinical characterization of PGAD/GPD symptoms (including quality, progression, severity, and location of symptoms as well as influences on the symptoms by analyzing trigger and relieving factors); (2) sexual, gynecological, urological, neurological, and psychiatric aspects; (3) assessment of existing comorbidities; (4) collection of diagnostical and non-pharmacological therapeutic approaches. All measures were collected during consultations in a specialized sexual medicine outpatient clinic conducted by 2 independent clinicians. Most data were obtained through self-report, complemented by standardized questions integrated into each consultation. (1) **Symptom Quality**. Patients were asked open-ended questions about the nature of their symptoms, followed by prompts addressing specific sensory qualities (eg, tingling, itching, burning, pressure, contractions, hypersensitivity, pain, or a sensation of imminent orgasm). **Symptom Progression and Severity**. Symptom course was assessed using 4 items describing continuous versus intermittent symptom patterns. Symptom intensity was rated on a numeric rating scale (NRS) (0–10). **Symptom Location and Radiation**. Patients identified the primary genital location of symptoms and potential radiation patterns typical of GPD (buttocks, legs, or back). **Triggering and Relieving Factors**. Open-ended questions explored potential triggers and factors alleviating symptoms. **Possible coinciding factors with PGAD onset.** Patients self-reported possible reasons for the onset of symptoms. (2) **Sexual and gynecological history**. Sexual and reproductive symptoms were assessed through questions on sexual impairment, dyspareunia, sexual experience, menstrual problems, menopausal status, parity, and hormonal contraception. **Urological history.** Urological symptoms were explored regarding overactive bladder (OAB), urinary urgency, and dysuria. **Neurological history**. Neurological symptoms included restless legs-like sensations or other abnormal sensations (eg, paresthesia, tingling). **Psychiatric history.** Psychiatric and trauma-related aspects were assessed by asking about experiences of psychological or physical trauma and possible family history of PGAD/GPD. (3) **Comorbidities**. Psychiatric and somatic comorbidities were assessed through open-ended questions obtained during the clinical history. This question captured possible yet unidentified comorbidities listed under section 2). (4) **Diagnostic measures and therapeutic interventions.** Patients reported prior diagnostic measures and therapeutic interventions. Common PGAD/GPD-related procedures were systematically reviewed, including MRI (head, pelvis, spinal cord), neurophysiological testing, gynecological, and urological examinations. Regarding therapeutic approaches, physiotherapy, psychotherapy, psychopharmacotherapy, complimentary methods, and invasive procedures were reviewed. Treatment benefit was assessed using a closed question: “Was the intervention symptom-relieving for you?” (yes/no). For the purposes of the study, responses to open-ended questions were subsequently categorized and summarized.

### Data analysis

All statistical analysis were conducted using SPSS Statistics Version 27 (IBM Corporation, Armonk, NY, USA). Descriptive statistics were calculated and are reported as mean (*M*) and standard deviation (± *SD*).

## Results

### Clinical characterization of PGAD/GPD

*Demographics:* In total, n = 92 subjects were included in the analysis, of which n = 86 were women and n = 6 were men. On average, PGAD/GPD patients were 43 years old (*M* = 42.65 ± 16.74 years; *min* = 9, *max* = 83). The mean age of the onset of the symptoms was 32 years (*M* = 31.65 ± 17.87 years; *min* = 1, *max* = 76); *Md* = 29.00 years). PGAD/GPD patients had an average age of 39 years (*M* = 39.18 ± 16.54 years; *min* = 7, *max* = 78) at the time of the first consultation at our clinic and attended the medical consultation about 3 times, either in person, by video consultation or by telephone (M = 3.33 ± 6.04; min = 1, max = 45). For more demographic information see Table 1.

**Table 1:** Sociodemographic factors.

Variable	PGAD/GPD	
Demographic (n = 92)	%	M ± SD/n
Female sex	93.5	86
Male sex	6.5	6
Age		42.65 ± 16.74
Age of symptom-onset		31.65 ± 17.87
Age of first consultation		39.18 ± 16.54
Employment status (n = 87)		
Unemployed	13.8	12
In training	19.5	17
Retired	14.9	13
Employed	51.7	45
Familial (n = 84*)*		
Marital status		
Unmarried/Relationship	38.1	32
Married	27.4	23
Unmarried/Single	29.8	25
Divorced	4.8	4

Sociodemographic factors. Abbreviations and symbols: GPD, genito-pelvic dysesthesia; PGAD, persistent genital arousal disorder; M, Mean; SD, standard deviation.

### Symptom severity

At the first occurrence of PGAD/GPD symptoms, patients rated their symptoms using the NRS—ranging from 0 (no symptoms at all) to 10 (worst symptoms ever experienced)—with an average rating of almost 7 (*M* = 6.78 ± 2.06; *min* = 2, *max* = 10, n = 73). Over the entire period of symptoms, symptom severity was estimated as *M* = 6.24 ± 1.65 on the NRS (*min* = 1, *max* = 9, n = 76). The most severe symptoms experienced by patients averaged with *M* = 8.26 ± 1.79 (*min* = 2, *max* = 10, n = 78), while the least symptomatic episodes averaged at a mean of *M* = 3.84 ± 2.52 (*min* = 0, *max* = 10, n = 77). More than half of the patients (62.3%) reported an average of at least 5 points on the NRS scale.

### Progression of symptoms

More than half of the patients (47 of 92) stated to have continuous symptoms with slight fluctuations, 19 had continuous symptoms with symptom attacks, 17 had symptom attacks with symptom-free intervals, and 9 of them had symptom attacks with symptoms in between. Twenty (23.5%, n = 85) PGAD/GPD patients indicated a circadian course of their symptoms. In individual cases, connections to the menstrual cycle were mentioned in the sense of an increase in symptoms during ovulation, but also during menstruation (see also trigger factors, Table 2).

**Table 2:** PGAD/GPD-related characteristics.

Variable		PGAD/GPD patients (n = 92)		
	%	n	Mean	SD
NRS—first occurrence of symptoms [0-10]			6.78	2.06
NRS—entire period of having symptoms [0-10]			6.24	1.65
NRS—most severe symptoms ever [0-10]			8.26	1.79
NRS—least severe symptoms ever [0-10]			3.84	2.52
Progression of symptoms				
Continuous symptoms with slight fluctuations	51.1	47		
Continuous symptoms with symptom attacks	20.7	19		
Symptom attacks, symptom-free in between	18.5	17	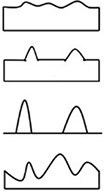	
Symptom attacks, symptoms in between	9.8	9		
Circadian course of symptoms	23.5	20/85		
Symptom quality				
Tingling	60.9	56		
Pain	30.4	28		
Unpleasant sensation by tight clothes	26.1	24		
Pulsating	23.9	22		
Pressure	19.6	18		
Contractions	16.3	15		
Burning	15.2	14		
Stabbing	12.0	11		
Hypersensitivity	12.0	11		
Tension	8.7	8		
Electrifying sensations	6.5	6		
Feeling of warmth	5.4	5		
Itch	4.3	4		
Feeling of being on the verge of an orgasm	26.1	24		
Spontaneous swelling of the genitals	52.5	42		
Extraordinary lubrication	32.9	25		
Spontaneous orgasms	30.4	28		
Trigger factors				
Mental stress	54.0	47		
Sitting	36.8	32		
Driving a bus/care/train	31.0	27		
Wearing tight clothes	29.9	26		
Vibration	29.9	26		
Being touched	20.7	18		
Lying down	18.4	16		
Sexual intercourse	18.2	16		
Menstruation	13.8	12		
Masturbation	12.6	11		
Rest	11.5	10		
Riding a bicycle	11.5	10		
Physical exercise	9.2	8		
Warmth	9.2	8		
Urinary urge	8.0	7		
Erotic material	8.0	7		
Going to the toilet	6.9	6		
Cold	4.6	4		
Alcohol use	2.3	2		
Caffeine consumption	1.1	1		
Reduction of nicotine use	1.1	1		
Memories of a past traumatic experience	1.1	1		
Relieving factors				
Distraction	38.9	35		
Physical exercise	33.3	30		
Masturbation	25.6	23		
Heat/warmth	24.4	22		
Relaxation	20.0	18		
Walking	22.2	20		
Cooling	17.8	16		
Sexual intercourse	17.8	16		
Physical pressure	14.4	13		
Lying down	11.1	10		
Pressure relief	6.7	6		
Going to the toilet	5.6	5		
Alcohol use	2.2	2		
Urinary urge	2.2	2		
Taping of the affected areas	1.1	1		

PGAD/GPD-related characteristics: The sums of the main item radiation differ from the sums of the subitems, because the patients made several statements regarding the individual subitems. Abbreviations and symbols: GPD, genito-pelvic dysesthesia; PGAD, persistent genital arousal disorder; NRS, numeric rating scale; SD, standard deviation.

### Localization

Regarding the exact symptom localization, data from n = 84 female patients was available. Seventy-one (84.5%) patients named the clitoris as the main symptom localization. Forty-seven (56.0%) named the vagina, 39 (46.4%) the vaginal opening, 27 (32.1%) the labia, 17 (20.2%) the mons pubis and the pelvic floor, 12 (14.3%) the urethra and the anus, 6 (7.1%) the perineum and dam region, and 5 (6.0%) the mammillae.

Regarding symptom localization in men with PGAD/GPD, n = 6 data sets were available. Parallel to the female subjects, the glans penis was named as the most frequent symptom localization by 5 subjects (83.3%), whereas the frenulum was named separately by 4 patients (66.7%). The corpus per se was named 3 times (50.0%). Two (33.3%) listed the testicles and the pelvic floor, 1 (16.7%) the perineum, dam region, and urethra and none of the patients listed the anus as a symptom localization. Forty-six of 90 subjects (51.1%) reported an extragenital manifestation of the symptoms. Nineteen (21.1%) described having symptoms in the legs, 14 (15.6%) in the bladder, 11 (12.2%) in the buttock, 7 (7.8%) in the back, 6 (6.7%) in the abdominal region, 5 (5.6%) in the breasts, 4 (4.4%) in the groin area, and 2 (2.2%) in the arms and in the whole body.

### Symptom quality

Fifty-six (60.9%) reported tingling or prickling sensations. Twenty-eight (30.4%) stated pain as the main quality of the symptoms. Twenty-four patients (26.1%) felt unpleasant sensations when the region was touched by tight clothing. Twenty-two (23.9%) named pulsating, 18 (19.6%) pressure, 15 (16.3%) contraction, 14 (15.2%) burning, 11 (12.0%) stabbing, and hypersensitivity, 8 (8.7%) tension, and 6 (6.5%) electrifying sensations. Five (5.4%) had a feeling of warmth and 4 (4.3%) felt an itch. According to the definition of PGAD, 24 (26.1%) felt permanently being on the verge of an orgasm. Patients also presented with high rates of swelling of the genitals (n = 42, 52.5%), spontaneous orgasms (n = 28, 30.4%), and extraordinary high lubrication (n = 25, 32.9%).

### Trigger and relieving factors

A total of 85 out of 88 patients (96.6%) indicated the presence of specific trigger factors. In total, 22 different trigger factors were identified. Most frequently reported trigger factors were mental stress (54.0%, n = 47), sitting (36.8%, n = 32), riding in a bus, car or train (31.0%, n = 27), wearing tight clothes, and vibration (each 29.9%, n = 26). Eighty-one out of 90 patients (90.0%) were able to report relieving factors, which amounted to a total of 15 different factors. Relieving factors were mainly distraction (38.9%, n = 35), physical exercise (33.3%, n = 30), masturbation (25.6%, n = 23), warmth/heat (24.4%, n = 22), and relaxation (20.0%, n = 18). For details of PGAD/GPD-related characteristics as well as trigger and relieving factors, see Table 2.

### Possible reasons for onset of PGAD/GPD symptoms

*Self assessment:* For almost half of the patients (n = 41), the etiology remained completely unknown. Nevertheless, some possible connections were mentioned, which were divided into the following 6 categories: (1) drug related influences, (2) psychological influences/traumatic experiences, (3) urogenital infections, (4) gynecological/hormonal influences, (5) lumbar/pelvic issues due to trauma or strain, (6) unknown. For more information, see Supplementary Appendix Table S1.

### Sexual, gynecological, urological, neurological, and psychiatric aspects of PGAD/GPD

**Figure 1:**
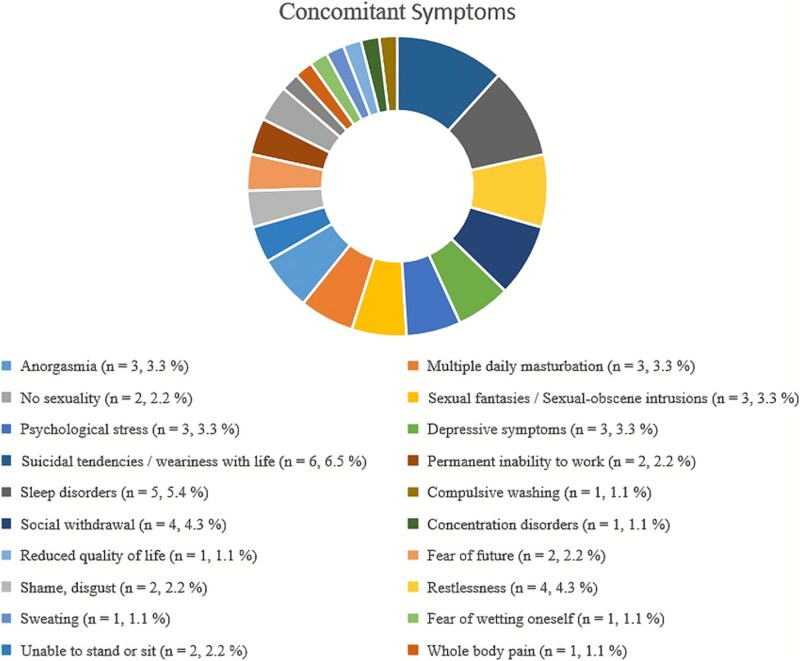
Concomitant symptoms in PGAD/GPD patients.

Over 73% (53 out of 72 available data) complained about impaired sexuality. In 10 patients, the symptoms were accompanied by pain during sex, ie, dyspareunia. One of our patients did not report any sexual experience. Fourteen patients reported menstrual problems (especially dysmenorrhea), whereby 2 have not yet been exposed to menarche. Twenty-four subjects were in the menopause. Thirty-six gave birth to either 1, 2 or 3 children, and 20 patients used hormonal contraception. More than 45% (40 of 88) stated the symptoms of OAB and 52.3% (46 of 88 available data) indicated increased urinary urge. About 23.8% (19 of 80 available data) reported dysuria. More than 33% (31 of 92) stated RLS. There was no significant relationship between OAB and RLS (Fisher’s exact test (N = 88), *p* = 0.65). With regard to traumatic experiences, 12 patients stated that they had experienced psychological trauma in childhood, adolescence, or adulthood. Seven patients reported physical trauma in the form of accidents. A small number of patients (4 out of 71 available data) reported a positive family history of PGAD/GPD. Other various individual concomitant symptoms can be found in Figure 1.

### Comorbidities

Almost 70% (64 of 92) had comorbid psychiatric diagnoses. The available data did not allow an accurate assessment of whether it was a lifetime or current comorbidity in each case. Half of the patients reported a depressive disorder and over one third stated suffering from an anxiety disorder. The majority also suffered from decreased sleep quality, ie, sleeping disorders. About 75.0% (n = 69) had somatic comorbidities, of which 30.4% were neurological diseases such as herniated discs, polyneuropathy, spastic hemiparesis, neuroborreliosis, migraine and tension headaches, epilepsy, and narcolepsy. In addition, almost 28% of the cases had gynecological comorbidities in the form of breast cancer, hysterectomy, cervical dysplasia, ovarian cysts, endometriosis, or fibroids. About 38.0% suffered from previous internal diseases, such as coronary heart disease, arterial hypertension, rheumatism, bronchial asthma, COPD, hypo- and hyperthyroidism, and type II diabetes mellitus. Almost 20% had surgical comorbidities, including osteoarthritis, scoliosis, colorectal carcinoma, diverticulosis, hernias, intestinal abscesses, and polyps. Urological diseases (16.3%) included urinary bladder dysfunction, bladder- or kidney cysts. A small percentage (8.7%) reported dermatological diseases, including melanoma, psoriasis, neurodermatitis, and mycoses. For further information to comorbid diagnosis, see Table 3.

**Table 3:** Psychiatric and somatic comorbidities in PGAD/GPD patients.

Comorbidity		
	n = 92	%
**Psychiatric comorbidity**	64	69.6
Sleeping disorder	54	58.7
Depressive disorder	46	50.0
Anxiety disorder	29	31.5
Obsessive-compulsive disorder	11	12.0
PTSD	8	8.7
Somatization disorder	7	7.6
Addiction disease	7	7.6
ADHD	5	5.4
Eating disorder	5	5.4
Personality disorder	3	3.3
Bipolar disorder	1	1.1
Schizophrenia	1	1.1
Hypochondria	1	1.1
**Somatic comorbidity**	69	75.0
Neurological diseases	28	30.4
Gynecological diseases	24	27.9
Internal diseases	35	38.0
Dermatological diseases	8	8.7
Surgical diseases	18	19.6
Urological diseases	15	16.3

Psychiatric and Somatic Comorbidities in PGAD/GPD patients. The sums of the subitems may differ from the sums of the total numbers and percentages, because patients often suffered from more than one disease out of each category. Abbreviations: ADHD, attention deficit hyperactivity disorder; GPD, genito-pelvic dysesthesia; PGAD, persistent genital arousal disorder; PTSD, post traumatic stress disorder.

### Diagnostical and non-pharmacological therapeutic approaches

A total of 82 out of 92 patients underwent various diagnostic tests to determine the cause of PGAD/GPD. Of these, gynecological examinations for PGAD were performed in over 65% of cases. An MRI of the spine was performed in over 50% of cases, an MRI of the pelvis in exactly 50% of cases and cranial MRI in almost 45% of the cases. Around 36% underwent measurement of nerve conduction velocities, 27% underwent urological examinations, and in 14.1% an EEG was performed. About 13% underwent endocrinological examinations. In rare cases, an EMG (6.5%), a sleep laboratory analysis (5.4%), a spinal tap (5.4%), or a duplex examination of the pelvic veins (1.1%) were performed. To detect any abnormalities in the respective diagnostic examinations, please refer to Table 4. Detailed diagnostic findings were not available due to the retrospective data collection. In a single case, venous congestion was treated surgically, resulting in partial remission of the symptoms.

**Table 4:** Diagnostic approaches relating PGAD/GPD symptoms.

Diagnostic instrument	Completed	Abnormal finding
	n/%	n/%
Any diagnostic regarding PGAD/GPD	82/89.1	
Gynecological	60/65.2	10/16.7
MRI (thoracic and lumbar spine)	47/51.1	24/51.1
MRI (pelvis)	46/50.0	26/56.5
MRI (cranial)	41/44.6	1/2.4
Nerve conduction velocities	33/35.9	5/15.2
Urological	25/27.2	11/44.0
EEG	13/14.1	2/15.4
Endocrinological	12/13.0	2/16.7
EMG	6/6.5	2/33.3
Sleep laboratory	5/5.4	4/80.0
Spinal tap	4/4.3	1/25.0
Duplex sonography	1/1.1	0/0.0

*Diagnostic approaches relating PGAD/GPD symptoms*. Abbreviations: EEG, electroencephalography, EMG, electromyography; GPD, genito-pelvic dysesthesia; MRI, magnetic resonance imaging; PGAD, persistent genital arousal disorder. The sums of the subitems may differ from the sums of the total numbers and percentages, because in multiple cases several diagnostical approaches were conducted for the patients with PGAD/GPD.

Non-pharmacological based therapeutic approaches were utilized by 58 (63.0%) patients. Those included psychotherapy (40.2%), physiotherapy (23.9%), pelvic floor exercises (19.6%), transcutaneous electrical nerve stimulation (TENS) (12.0%), relaxation techniques (9.8%), non-medical/alternative practitioners (10.9%), pain therapy (9.8%), sexual therapy (7.6%), homeopathic medicine (6.5%), acupuncture (5.4%), yoga (3.3%), using a seating ring (1.1%), biofeedback (1.1%), and nutritional change (1.1%). About 1.1% used transcranial magnetic stimulation and electroconvulsive therapy. Regarding invasive procedures, in 5.4% a healing attempt with a pudendal block was tried. In 1.1% a neurosurgical procedure by removal of Tarlov cysts and varicose vein surgery were performed. To evaluate the effectiveness of the various therapeutic approaches, see Table 5.

**Table 5:** Non-medication-based therapeutic approaches relating PGAD/GPD symptoms.

Non-pharmacological therapy attempt	Completed n = 92	Provided relief	Did not provide relief	Missing data regarding effectiveness
	n/%	n/%	n/%	n
**Any non-medication based therapeutic attempt**	58/63.0			
**Non-invasive therapy attempt**				
Psychotherapy	37/40.2	17/68.0	8/32.0	12
Physiotherapy	22/23.9	10/55.6	8/44.4	4
Pelvic floor exercises	18/19.6	9/50.0	9/50.0	0
Transcutaneous electrical nerve stimulation	11/12.0	2/20.0	8/80.0	1
Alternative practitioner	10/10.9	1/10.0	9/90.0	0
Relaxation techniques	9/9.8	3/42.9	4/57.1	2
Pain therapy	9/9.8	5/55.6	4/44.4	0
Sexual therapy	7/7.6	2/40.0	3/60.0	2
Homeopathic medicine	6/6.5	1/16.7	5/83.3	0
Acupuncture	5/5.4	1/25.0	4/75.0	1
Yoga	3/3.3	2/66.7	1/33.3	0
Transcranial magnetic stimulation	1/1.1	0/0.0	1/100.0	0
Electroconvulsive therapy	1/1.1	0/0.0	1/100.0	0
Biofeedback	1/1.1	1/100.0	0/0.0	0
Nutritional change	1/1.1	0/0.0	0/0.0	0
Seating ring	1/1.1	?	?	1
**Invasive therapy attempt**				
Pudendal block	5/5.4	3/60.0	2/40.0	0
Neurosurgical procedure (Tarlov cyst)	1/1.1	1/100.0	0/0.0	0
Varicose vein surgery	1/1.1	1/100.0	0/0.0	0

*Non-medication-based therapeutic approaches relating PGAD/GPD symptoms. Non-invasive and invasive therapy attempts.* The sums of the subitems may differ from the sums of the total numbers and percentages, because in multiple cases several therapeutic approaches were conducted for the patients with PGAD/GPD. Missing data on the success of a therapy attempt resulted from the fact, that it was sometimes not possible to follow the course of treatment, as there were no further consultations. Abbreviations: GPD, genito-pelvic dysesthesia; PGAD, persistent genital arousal disorder.

## Discussion

### Summary and interpretation of results

This is the first registry study covering a considerable number of 92 patients affected by PGAD/GPD, capturing a wide range of clinical characteristics including symptom quality, progression, severity, and location as well as trigger and relieving factors. Furthermore, sexual, gynecological, urological, neurological, and psychiatric investigations were conducted.

In line with the results from our previous study,^32^ our patients were 32 years on average at manifestation of PGAD/GPD and first came to our consultation at a mean age of 39 years. This indicates a 7-year gap between the onset of symptoms and the diagnosis, emphasizing a lack of knowledge about the disease among practitioners. However, the patient’s sense of shame may considerably influence the delay in consulting a doctor when symptoms occur, and experiences of both shame and guilt should be further explored in future studies.^44–46^ Interestingly, the men in our cohort were younger both at the onset of symptoms (*M*_men_ = 19.8 vs. *M*_women_ = 32.5 years) and at first presentation in our consultation (*M*_men_ = 30.5 vs. *M*_women_ = 39.8 years). This suggests that more gender-specific examinations may be relevant in the future. It is important to point out that the number of male patients was significantly lower in our sample, limiting interpretation of results.

Symptoms were permanently present in over 70% of patients (either undulating or characterized by symptom attacks) and the severity of symptoms was on average never less than 4 on the Numeric Rating Scale (NRS) and reached on average a worst severity of over 8 on the NRS. Thus, in most cases, symptoms appear to be always present, leaving the patients with little respite from their symptoms, and thereby significantly increasing their suffering. Furthermore, we were able to confirm the main localization of the symptoms to be the clitoris. With regard to the localization of symptoms in men, preliminary findings have been obtained which indicates, that the glans penis is typically identified as the primary localization. Almost 1/3 (27.8%, n = 25) of the extragenital manifestations were located in the legs or buttocks, which supports the newly introduced term of GPD, including also lower extremity dysesthesia. The higher rates in spontaneous swelling of the genitals, extraordinary lubrication, and spontaneous orgasms support the definition of PGAD as a condition, which is usually not associated with sexual desire or sexual stimuli. In addition, it lends weight to the enormous suffering that patients endure in their daily lives. There is a broad variety in trigger and relieving factors. Even though they are very individual for most patients, we found more frequent and less frequent factors, that can help clinicians to find the right treatment and to understand the patients better.

The high interindividual variability also suggests different causes for PGAD, with theories about pathophysiology focusing on deficits in the central (eg, mental stress, medication) or peripheral (eg, sitting, wearing tight clothes) nervous system. The detailed self-assessment of the possible causes of the disease and the successful classification into 5 different main areas (urogenital influences, gynecological influences, lumbar/pelvic issues, drug-related influences, and psychological influences) suggest a great value of the proposed systematic examination of the 5 regions of the ISSWSH consensus statement (end organ, pelvis and perineum, cauda equina, spinal cord, and brain and assessing psychological factors).^3^ Most of the named subjective causes were related to therapy or withdrawal of antidepressive medication (withdrawal of antidepressants: n = 16, 17.4%; therapy with antidepressants: n = 6, 6.5%). For detailed pharmacological influences on our cohort, see Supplementary Appendix Table S2, based and redrawn on Kruger et al..^40^ Four of our 92 patients reported a positive family history for PGAD/GPD. This observation may suggest a potential genetic contribution to the disorder. An X-linked recessive inheritance pattern could be hypothesized, given the rarity of cases in males. However, since this information is based solely on self-reported family history and potential cases within families may have remained undiagnosed. This assumption remains speculative. Future studies employing genomic assays and RNA analyses in larger patient cohorts will be essential to clarify possible hereditary components.

An extraordinarily high heterogeneity in the manifestations of accompanying symptoms was observed. Of particular note are the increased difficulties with one’s own sexuality as well as increased psychological symptoms up to weariness with life or even suicidal thoughts. The high rates of impaired sexuality and comorbid psychiatric diagnoses (both over 2/3) observed in our patients may reflect a considerable burden associated with PGAD. These findings suggest that PGAD could negatively influence quality of life, which is consistent with our previous findings.^32^ While our previous study did not reveal any differences in suicidality, the present study notably suggests—consistent with findings from other studies^10^^,^^13^^,^^35^^,^^36^—that patients with PGAD may have a higher rate of suicidal thoughts (see concomitant symptoms illustrated in Figure 1). This observation could be due to the larger sample size in the current study and again could emphasize the high mental burden resulting from PGAD. Remarkably, more than 45% stated the symptoms of OAB, more than half of the subjects indicated increased urinary urge, and more than one third had restless legs symptoms, supporting the hypothesis that PGAD may be a variant of RLS as well as OAB. Both syndromes are discussed in the context of hyperexcitability of the dopaminergic system. The possible relationship between these syndromes and the associated dopamine deficiency could be one reason for the increased rates of urinary urgency and RLS in women with PGAD.^25^ Another possible explanation for this finding is the overlap between bladder and genital function in terms of their spinal representation (S2-S4). Nevertheless, there are very few reports of successful and sustained relief of PGAD symptoms through L-Dopa or dopamine agonists. For example, Ari and Sahin (2024) described a successfully managed case by using pramipexole^47^ and Lynn (2021) and her colleagues were able to improve PGAD symptoms in a 36 year old woman by low dose pramipexole, but not with higher doses.^48^ However, a relationship between OAB and RLS could not be found in our patients and administration of dopaminergic drugs did not lead to decreased symptoms.^40^ Diagnostic procedures covered urological, gynecological, and neurological examinations as well as MRI of brain, spinal cord, and pelvis. Non-pharmacological therapeutic approaches included a wide range of invasive and non-invasive procedures, for example physiotherapy, psychotherapy, TENS, neurosurgical procedures, or pudendal block. Psychotherapy and physiotherapy appeared to be some of the more promising approaches. These results should be interpreted with caution, as they are based on patient self-reports without standardized measures or additional variables to infer treatment effectiveness. Moreover, part of the sample (28.3%, n = 26) had already been examined in our previous study (iPGAD study^32^), which should be considered when interpreting overlapping findings. The multitude of diagnostic and therapeutic approaches suggests a desperate search for improvement but also indicates that this condition is very likely multifactorial in its origin.

### Limitations and opportunities

This registry study, conducted within a clinical, non-interventional, and retrospective framework, provides valuable insights from a large patient population. The data were collected during routine sexual medicine consultations, following a generally consistent though not fully standardized workflow.

However, several limitations must be considered. First, the retrospective, observational design, and reliance on patient self-reports limit the ability to draw causal inferences and rigorously establish treatment effectiveness. Data quality issues, including completeness and accuracy, may further affect validity.

Second, the study sample is heterogeneous and non-selected, with limited baseline data. The inclusion of healthy control participants and patients with relevant comorbidities would have been important to better characterize PGAD/GPD features and allow for a more detailed differential analysis.

Third, regarding treatment-related observations, some patients reported benefits from psychotherapy, physiotherapy, and psychopharmacotherapy (as described earlier in the manuscript); however, Table 5 shows that individual patients often received multiple interventions. Thus, the study design does not allow for conclusions about the effectiveness of any single therapeutic approach and these observations should be interpreted with caution.

Finally, unlike randomized controlled trials, which often lack real-world representativeness due to strict population selection and controlled scenarios, registry studies reflect actual clinical treatments and interventions. Nonetheless, ensuring methodological rigor in design and data collection remains crucial to maximize reliability and clinical relevance.

## Conclusion

Based on our findings, there is still no evidence for a clear causal relationship with a specific finding. Therefore, the authors still see the need for systematic investigation of patients with PGAD/GPD, as proposed by the International Society for the Study of Women’s Sexual Health. Under the assumption, that PGAD/GPD is a multifactorial condition, the goal should be the implementation of a comprehensive diagnostic and treatment strategy to guide clinicians both in diagnostics and treatment of PGAD/GPD. This strategy is based on 5 steps where each step reflects one part of the body, which shall be investigated: (1) end organ, (2) pelvis and perineum, (3) cauda equina, (4) spinal cord, (5) brain. Additionally, a thorough patient history should be obtained, covering symptomatology, trigger and relieving factors, medication history, and mental health status.^3^ In particular, general practitioners, psychiatrists, psychotherapists, gynecologists, urologists, and neurologists play a crucial role in recognizing and managing PGAD/GPD. Health care professionals should be aware of PGAD symptoms, assess key clinical feature—including trigger and relieving factors—and systematically diagnose and treat affected patients. Despite the lack of knowledge about the pathology of PGAD/GPD, it should be emphasized that patients can still be treated successfully, particularly with psychotherapy, physiotherapy, and psychopharmacotherapy. For helpful pharmacotherapy options, please refer to the results of Krüger et al. (2024) and also see Supplementary Appendix Table S3 (based on^40^). Researchers, clinicians and patients agree, that PGAD is a severely distressing and life-impairing disease.^3^ Due to the continued lack of controlled clinical studies, future research should focus on larger sample sizes, more detailed clinical assessments, and well-designed controlled studies to improve our understanding of PGAD/GPD. Furthermore, additional investigations using larger samples and advanced techniques are needed to determine how and where the peripheral or central nervous systems are involved in the pathophysiology of PGAD/GPD.

## Supplementary Material

Appendix_A_Table_6_qfaf106

Appendix_B_Table_7_qfaf106

Appendix_C_Table_8_qfaf106

## Data Availability

The datasets generated during and/or analyzed during the current study are available from the corresponding author on reasonable request.
