# Supplementary material for: Clinical characterization and management of persistent genital arousal disorder/genito-pelvic dysesthesia (PGAD/GPD): a registry study
Source: Sex Med. 2026 Jan 31;14(1):qfaf106. doi: 10.1093/sexmed/qfaf106 (PMC12860204; doi:10.1093/sexmed/qfaf106)
Supplement: Appendix_A_Table_6_qfaf106 [file appendix_a_table_6_qfaf106.docx]

| **Drug related influences** | **Psychological influences / Traumatic experiences** | **Urogenital infections** | **Gynaecological / hormonal influences** | **Lumbar / pelvic issues due to trauma or strain** | **Unknown** |
| --- | --- | --- | --- | --- | --- |
| Discontinuation of SSRI  (sertraline (*n* = 3), paroxetine (*n* = 3), fluoxetine (*n* = 1), escitalopram  (*n* = 1), fluvoxamine (*n* = 1))  (∑*n* = 9) | Sexual abuse at the age of 9 years (*n* = 1) | Urinary tract infection  (∑*n* = 9) | Sexual intercourse (*n* = 1) | Prolonged sitting on the train (*n* = 1) | Unknown or sudden beginning  (*n* = 42) |
| Discontinuation of NSMRI (opipramol (n = 1), trimipramine  (n = 1), amitriptyline (*n* = 1))  (∑*n* = 3) | Sexual assault by father (*n* = 1) | fungal infections  (∑*n* = 2) | ovarian cyst (*n* = 1) / after multiple operations of ovarian cysts (*n* = 1)  (∑*n* = 2) | Birth of child (*n* = 1) |  |
| Discontinuation of NaSSA (mirtazapine) (∑*n* = 2) | Since childhood (*n* = 1) |  | Menarche  (∑*n* = 2) | Bicycle tour (*n* = 1) |  |
| Discontinuation of chlorprothixene  (*n* = 1) | Divorce from ex-husband (*n* = 1) |  | After one very heavy period (*n* = 1) | Fall on coccyx after skiing accident (*n* = 1) |  |
| Discontinuation of pregabalin  (∑*n* = 2) | Sex workshop  (*n* = 1) |  | Hysterectomy (*n* = 1) | Active lumbar spine and pelvic exercises (*n* = 1) |  |
| Discontinuation of SNRI (venlafaxine (*n* = 1), duloxetine  (*n* = 1)) (∑*n* = 2) |  |  | Using TENS intravaginally after having a bladder prolapse (*n* = 1) | BS prolapse L4/L5 (*n* = 1) |  |
| Therapy with SSRI  (citalopram (n = 1),  fluoxetine (*n* = 1)) (∑*n* = 2) |  |  | Menopause (*n* = 1) | “twisting” of the hip with “cracking” (*n* = 1) |  |
| Therapy with Duloxetine (*n* = 1) |  |  |  |  |  |
| Therapy with opipramol (*n* = 1) |  |  |  |  |  |
| Therapy with amitriptyline (*n*= 1) |  |  |  |  |  |
| Therapy with quetiapine (*n* = 1) |  |  |  |  |  |
| Therapy with anticonvulsants (*n* = 1) |  |  |  |  |  |
| Therapy with trazodone (*n* = 1) |  |  |  |  |  |
