# Supplementary material for: Clinical characterization and management of persistent genital arousal disorder/genito-pelvic dysesthesia (PGAD/GPD): a registry study
Source: Sex Med. 2026 Jan 31;14(1):qfaf106. doi: 10.1093/sexmed/qfaf106 (PMC12860204; doi:10.1093/sexmed/qfaf106)
Supplement: Appendix_B_Table_7_qfaf106 [file appendix_b_table_7_qfaf106.docx]

Appendix B: Table 7. Drugs that may induce PGAD symptoms due to discontinuation, reduction or intake of the drug; redrawn and modified according to Krüger, Köhne & Kümpers, 2024

| Drug class | Name | Withdrawal of drug (N) | Therapy with drug (N) | Reducing drug (N) |
| --- | --- | --- | --- | --- |
| Antidepressants |  |  |  |  |
| SSRI |  | 1 | 0 | 1 |
|  | Fluoxetine | 2 | 1 | 0 |
|  | Paroxetine | 2 | 0 | 0 |
|  | Citalopram | 0 | 1 | 0 |
|  | Sertraline | 2 | 0 | 0 |
|  | Escitalopram | 1 | 0 | 0 |
| SNRI |  |  |  |  |
|  | Duloxetine | 0 | 1 | 1 |
|  | Venlafaxine | 2 | 0 | 0 |
| NSMRI |  |  |  |  |
|  | Amitriptyline | 1 | 1 | 0 |
|  | Trimipramine | 1 | 0 | 0 |
|  | Opipramol | 1 | 1 | 0 |
| NaSSA | Mirtazapine | 1 | 0 | 0 |
| Melatonine analoga | Agomelatine | 1 | 0 | 0 |
| Anticonvulsants |  | 0 | 1 | 0 |
|  | Pregabalin | 2 | 0 | 0 |
| Antipsychotics,  first generation | Chlorprothixene | 1 | 0 | 0 |
| Antipsychotics,  second generation | Quetiapine | 0 | 1 | 0 |
| Hormones | Progesterone / DHEA | 0 | 1 | 0 |

Table 7 – The groups *Withdrawal of drug, Therapy with* and *Reducing drug* mean, that PGAD was possibly triggered either during therapy, discontinuation or reduction of the respective medication. Some patients could only name the substance class and not the agent itself.
