# Supplementary material for: Clinical characterization and management of persistent genital arousal disorder/genito-pelvic dysesthesia (PGAD/GPD): a registry study
Source: Sex Med. 2026 Jan 31;14(1):qfaf106. doi: 10.1093/sexmed/qfaf106 (PMC12860204; doi:10.1093/sexmed/qfaf106)
Supplement: Appendix_C_Table_8_qfaf106 [file appendix_c_table_8_qfaf106.docx]

Appendix C: Table 8. Drugs that may influence PGAD symptoms,

*n* = 90; redrawn and modified according to Krüger, Köhne & Kümpers, 2024

| Drug class | Name | No. of subj. (%), N = 90 | Improved (N) | Worsened (N) | No effect (N) | Missing data (N) |
| --- | --- | --- | --- | --- | --- | --- |
| Antidepressants |  | 53 (58.9) |  |  |  |  |
| SSRI |  | 18 (20.0) |  |  |  |  |
|  | Fluoxetine | 2 (2.2) | 0 | 2 | 0 | 0 |
|  | Paroxetine | 2 (2.2) | 0 | 1 | 1 | 0 |
|  | Citalopram | 8 (8.9) | 1 | 2 | 5 | 0 |
|  | Sertraline | 6 (6.7) | 2 | 0 | 4 | 0 |
|  | Escitalopram | 3 (3.3) | 1 | 0 | 2 | 0 |
| SNRI |  | 40 (44.4) |  |  |  |  |
|  | Duloxetine | 40 (44.4) | 18 | 6 | 12 | 4 |
|  | Venlafaxine | 6 (6.7) | 3 | 1 | 2 | 0 |
| NSMRI |  | 14 (15.6) |  |  |  |  |
|  | Amitriptyline | 7 (7.8) | 1 | 1 | 3 | 2 |
|  | Trimipramine | 3 (3.3) | 0 | 0 | 3 | 0 |
|  | Opipramol | 4 (4.4) | 1 | 2 | 1 | 0 |
|  | Imipramine | 2 (2.2) | 1 | 0 | 1 | 0 |
|  | Clomipramine | 1 (1.1) | 1 | 0 | 0 | 0 |
|  | Doxepin | 1 (1.1) | 0 | 0 | 1 | 0 |
| NaSSA | Mirtazapine | 2 (2.2) | 1 | 0 | 1 | 0 |
| Melatonine analoga | Agomelatine | 2 (2.2) | 0 | 0 | 2 | 0 |
| NDRI | Bupropion | 1 (1.1) | 0 | 1 | 0 | 0 |
| Anticonvulsants |  | 38 (42.2) |  |  |  |  |
|  | Pregabalin | 31 (34.4) | 11 | 2 | 15 | 3 |
|  | Gabapentin | 13 (14.4) | 3 | 0 | 9 | 1 |
|  | Topiramate | 1 (1.1) | 0 | 0 | 1 | 0 |
| Antipsychotics,  second generation |  | 8 (8.9) |  |  |  |  |
|  | Clozapine | 1 (1.1) | 1 | 0 | 0 | 0 |
|  | Aripiprazole | 2 (2.2) | 2 | 0 | 0 | 0 |
|  | Quetiapine | 4 (4.4) | 0 | 0 | 3 | 1 |
|  | Amisulpride | 2 (2.2) | 0 | 0 | 2 | 0 |
|  | Sulpiride | 1 (1.1) | 0 | 0 | 1 | 0 |
| Anticholinergics | Trospium (Spasmex) | 6 (6.7) | 1 | 1 | 3 | 1 |
| Pseudoephedrine | Acetylsalicylic acid (ASS) | 1 (1.1) | 1 | 0 | 0 | 0 |
| Partial agonist at nicotinic acetylcholine receptor subtypes | Varenicline | 1 (1.1) | 0 | 0 | 1 | 0 |
| DOPA-Decarboxylase-inhibitor | Benserazide | 4 (4.4) | 0 | 3 | 1 | 0 |
| Benzodiazepines | Lorazepam, Oxazepam & others | 12 (13.3) | 8 | 0 | 3 | 1 |
| Toxins | Botulinum toxin | 6 (6.7) | 2 | 0 | 3 | 1 |
| Cannabinoids | Tetrahydrocanna-binol, Cannabidiol | 8 (8.9) | 7 | 0 | 1 | 0 |
| Sodium channel blocker | Lidocaine | 19 (21.1) | 4 | 1 | 13 | 1 |
| Opioids | Oxycodone, Tramadol & others | 17 (18.9) | 10 | 0 | 2 | 5 |
| Hypnotics | Z-drugs | 11 (12.2) | 6 | 0 | 1 | 2 |
| Muscle relaxants |  | 3 (3.3) |  |  |  |  |
|  | Methocarbamole | 2 (2.2) | 1 | 0 | 1 | 0 |
|  | Tizanidine | 1 (1.1) | 1 | 0 | 0 | 0 |
| Sexual hormones |  | 2 (2.2) |  |  |  |  |
|  | Chlormadinone | 2 (2.2) | 1 | 0 | 1 | 0 |
|  | Estradiol | 1 (1.1) | 1 | 0 | 0 | 0 |
| Antiandrogens | Cyproterone actetate (Androcur) | 2 (2.2) | 2 | 0 | 0 | 0 |

Table 8 – Improved PGAD = PGAD symptoms were improved by the respective medication. Worsened PGAD = PGAD symptoms were worsened by the respective medication. Multiple answers of each patient were possible. It is therefore possible that the sums of the substance classes (e.g. SNRI) do not correspond to the individual substances (e.g. sum of Duloxetine and Venlafaxine)
